# Melatonin-induced *Bacillus tequilensis* enhanced the disease resistance of *Camellia oleifera* against anthracnose by modulating cell wall and phenylpropanoid metabolism

**DOI:** 10.3389/fpls.2025.1593369

**Published:** 2025-06-09

**Authors:** Aiting Zhou, Huiqin Zhou, Ruiqi Peng, Di Liu, Jianrong Wu, Jia Deng, Fang Wang

**Affiliations:** ^1^ College of Forestry, Southwest Forestry University, Kunming, China; ^2^ Key Laboratory of State Forestry and Grassland Administration on Biodiversity Conservation in Southwest China, Southwest Forestry University, Kunming, China; ^3^ Key Laboratory for Forest Resources Conservation and Use in the Southwest Mountains of China, Ministry of Education, Southwest Forestry University, Kunming, China

**Keywords:** melatonin, *Bacillus tequilensis*, anthracnose, enzyme activity, transcriptome analysis

## Abstract

**Introduction:**

Anthracnose poses a significant threat to the sustainable development of the *Camellia oleifera* industry. In this study, we aimed to assess the efficacy of melatonin-induced *Bacillus tequilensis* DZY6715 (MT-DZY 6715) in controlling *C. oleifera* anthracnose caused by *Colletotrichum siamense*.

**Methods:**

The antifungal activity of MT-DZY6715 against *C. siamense* was systematically evaluated *in vitro* dual-culture assays and in planta infection trials. Furthermore, we analyzed the impact of MT-DZY 6715 on the anatomical features of *C. oleifera* leaves using histological sectioning. Additionally, transcriptome and enzymatic assays were employed to assess the expression of secondary metabolism-related genes and the activity of cell wall-degrading enzymes.

**Results:**

The application of MT-DZY 6715 effectively controls the growth of *C. siamense*, markedly reducing the incidence of *C. oleifera* anthracnose and delaying the spread of pathogens on the leaves. Furthermore, MT-DZY 6715 treatment enhanced leaf thickness, palisade mesophyll, as well as elevated the ratio of palisade mesophyll to spongy mesophyll (PS) and tissue compactness (CTR), while simultaneously decreasing tissue looseness (SR). Meanwhile, the leaf stomata undergo a reduction in size and a decrease in their openness. In addition, MT-DZY 6715 promoted the expression of secondary metabolism-related genes, such as PAL, CAL, Laccase, HCT, and CHI, and inhibited the activity of enzymes related to cell wall degradation (pectinase and cellulase), thereby coordinating and enhancing the accumulation of secondary metabolites and strengthening the mechanical properties of the cell wall.

**Discussion:**

The results of this study demonstrate that MT-DZY 6715 enhances the resistance of *C. oleifera* to anthracnose by modulating leaf morphology, regulating stomatal function, and promoting the accumulation of secondary metabolites. These findings provides a scientific foundation for deploying MT-DZY6715 as an eco-friendly alternative to chemical fungicides in anthracnose management.

## 1 Introduction

*Camellia oleifera*, widely known as the tea oil tree, is an evergreen shrub or small tree of the genus *Camellia* in the Theaceae family. It stands out as one of the four primary woody oil seed plants, earning the esteemed title of “Oriental Olive Oil” owing to its remarkable economic and culinary value (Yang et al., 2024). Additionally, *C. oleifera* is acclaimed for its medicinal benefits and holds vital ecological significance. However, as the cultivation area of *C. oleifera* expands, anthracnose has gradually emerged as a major obstacle to the industry’s development. Anthracnose, caused by *Colletotrichum* species (Hao et al., 2023; Jeyaraj et al., 2023), is characterized by its rapid spread, wide incidence, and high virulence, posing a grave threat to the health of *C. oleifera* (Chen et al., 2023). In the field, leaves infected by the anthracnose pathogen initially exhibit dark brown or yellowish-brown semicircular spots. As the disease advances, these spots gradually expand and turn into grayish-white patches adorned with black fruiting bodies (Jiang and Li, 2018). The damage of anthracnose is not limited to leaves, it also impacts fruits, branches, and other parts of the plant, ultimately leading to defoliation, fruit drop, and even the death of the entire plant (Muntala et al., 2020). These severe consequences not only significantly impact the yield and quality of *C. oleifera* but also result in huge economic losses.

In the face of this challenge, although traditional chemical control methods can provide some suppression of the disease, they also bring drawbacks such as pathogen resistance, chemical residues, and ecological impacts that cannot be overlooked ignored (Martinez et al., 2020; Xia et al., 2023). Thus, the pursuit of green, safe, and eco-friendly biological control measures has become a primary focus (Kumar Ahirwar et al., 2019). Microbial preparations are known for their low toxicity, minimal residue, and environmental friendliness (Ashraf et al., 2014), making them an ideal alternative to chemical pesticides for controlling diseases in the *C. oleifera* industry. Their application may promote the green and sustainable development of the *C. oleifera* industry, helping to achieve a harmonious balance of economic, ecological, and social benefits. *Bacillus* species is often regarded as ideal candidates for biological control due to their ability to enhance plant resistance by producing antagonistic compounds that inhibit pathogen growth and inducing systemic resistance, as reported in several studies (Fira et al., 2018; Miljaković et al., 2020; Ongena et al., 2007; Wang et al., 2018). However, the efficacy of *Bacillus* is inferior to that of chemical fungicides, so it is necessary to develop methods to improve its biological efficiency.

Melatonin (N-acetyl-5-methoxytryptamine, MT) is a versatile small molecule substance widely distributed in both animals and plants. It is a natural compound, serving as a potent antioxidant and free radical scavenger, playing a crucial role in enhancing plant disease resistance (Hernández-Ruiz et al., 2023). Bisquert et al. (2018) suggested under oxidative stress conditions, *Saccharomyces cerevisiae* treated with MT exhibited significantly modulated gene expression related to stress protection, leading to an increased cell viability of up to 35% compared to control group. Peter et al. (2024) discovered that MT and *Bacillus* sp. IPR-4 co-inoculation significantly enhanced soybean resistance to drought stress by promoting growth, optimizing nutrient absorption, regulating redox homeostasis, and upregulating drought-responsive genes. Furthermore, studies have confirmed that melatonin can enhance resistance in litchi fruit cell walls by modulating membrane lipid and energy metabolisms, thereby retarding browning, senescence, and lesion expansion caused by pathogens (Wang et al., 2020a; Zhang et al., 2021). Plant-produced secondary metabolites, including phenolics, flavonoids, and lignin, work together with the cell wall to construct a defensive system, while melatonin further promotes the synthesis of these disease-resistant metabolites, significantly boosting the plant’s disease resistance (Anjali et al., 2023). Additionally, Liu et al. (2019) reported that melatonin enhances tomato fruit resistance to *Botrytis cinerea* by modulating H_2_O_2_ levels and the jasmonic acid (JA) signaling pathway. MT enhances plants’ disease resistance by activating defense signaling pathways, such as ROS scavenging, MAPK activation, and crosstalk with SA/JA (Tiwari et al., 2022). Song et al. (2022) discovered that Melatonin improves the postharvest quality of eggplant fruits by inhibiting the activity and gene expression of cell wall-degrading enzymes (PME, PG, and Cel).

Currently, there is limited information available on the application of melatonin induced *Bacillus* sp. in enhancing disease resistance in *C. oleifera*, especially regarding the mechanisms underlying the induction of physical defense at both genetic and physiological levels. Thus, in this study, melatonin was employed to induce *B. tequilensis* DZY 6715 (MT-DZY 6715), with the aims of exploring: 1) the inhibitory effects of MT-DZY 6715 on *Colletotrichum siamense in vitro*; 2) the influence of MT-DZY 6715 on the structural characteristics of *C. oleifera* leaves; 3) the relevant disease-resistance genes through transcriptome analysis following MT-DZY 6715 treatment; and 4) the enzymatic activities associated with cell wall and phenylpropanoid metabolism in *C. oleifera* plants induced by MT-DZY 6715 treatment. The findings of this study offer novel insights into the molecular mechanisms that enhance disease resistance in *C. oleifera* through the induction of MT-DZY 6715.

## 2 Materials and methods

### 2.1 Plant, pathogen, bacterial strain and melatonin

Two-year-old *C. oleifera* seedlings (‘Changlin No. 53’) sourced from Yingjiang Linli Oil Tea Co., Ltd. in Yunnan, China, were housed in a greenhouse at Southwest Forestry University. The greenhouse conditions were maintained at a controlled temperature of 25°C and humidity of 80%, with regular irrigation using sterile water.

The pathogenic fungus, *C. siamense*, was isolated from *C. oleifera* leaves affected by anthracnose. Identification was confirmed through sequencing of the Internal Transcribed Spacer (ITS) region using universal primers ITS1 and ITS4. The pathogenic fungus was maintained on the slope of a Potato Dextrose Agar (PDA) medium at a temperature of 4°C for preservation.

The *B. tequilensis* DZY 6715 strain was isolated from *C. oleifera* and cultured in Luria-Bertani (LB) medium at the College of Forestry, Southwest Forestry University, located in Kunming, Yunnan Province, China.

MT (CAS: 73-31-4) was obtained from Merck (Sigma-Aldrich Shanghai Trading Co., Ltd.) and stored under 4°C conditions.

### 2.2 Preparation of MT-DZY 6715 suspension

Based on our previous research (Liu et al., 2023), an MT concentration of 50 μmol L^-1^ was selected. Regarding the determination of culture time, *B. tequilensis* DZY 6715 reaches its peak viable cell count and biofilm-forming capability after being cultured for 72 h when induced by MT at a concentration of 50 μmol L^-1^. Consequently, a culture period of 72 h was selected for further investigation.

### 2.3 Effect of MT-DZY 6715 against *C. siamense in vitro*

The antifungal test was conducted utilizing the plate confrontation method (Zhou et al., 2022). Five-day-old viable cultures of *C. siamense*, each with a diameter of 6 mm, served as the indicator pathogen and were placed at the center of 90 mm petri dishes filled with PDA medium. Treatment groups were established by injecting the following solutions 3 cm away from the center of each dish: (1) 50 μmol L^-1^ MT, (2) 72-h DZY 6715 suspension at a concentration of 1×10^7^ CFU mL^-1^, and (3) DZY 6715 suspension that had been induced with 50 μmol L^-1^ MT for 72 h (MT-DZY 6715), at a concentration of 1×10^7^ CFU mL^-1^. The control group consisted of petri dishes with the pathogen inoculated alone. All petri dishes were then placed in an incubator at 28°C. Adopted from Al Farraj and Elshikh (2023), the inhibition rate (R) was calculated was calculated as follows: R = (R1 - R2)/R1, where R1 and R2 represent the diameters of the pathogen colonies in the control and treatment groups, respectively. Each treatment was replicated three times, and the experiment was conducted twice.

### 2.4 Effect of MT-DZY 6715 against *C. siamense* on *C. oleifera*

Healthy, uniform, and mechanical damage-free *C. oleifera* seedlings were selected. The leaves were sanitized with 75% ethanol for 30 s, rinsed three times with sterile water. Then the selected seedlings following treatments with MT, DZY 6715, MT-DZY 6715, and sterile water as a control. Each seedling received 20 mL of the respective solution applied evenly with a spray bottle, ensuring no droplets fell off, and the leaves were allowed to dry before reapplying. Twenty-four hours post-inoculation, created on either side of the central vein of the leaves using a sterile inoculation needle. Subsequently,10 μL of conidial suspension of *C. siamense*, at a concentration of 1 × 10^5^ CFU mL^-1^ was applied to the wounded area of each leaf. The seedlings were cultivated at room temperature with a controlled humidity of 80%. Finally, the lesion size on the leaves and the incidence rate (M) were calculated based on the Xie et al. (2021) ‘s approach, as formulated:


M=(M1/M2)×100%

where M1 and M2 represent the number of infected leaves and the total number of leaves, respectively. Each treatment had nine replicates, and the experiment was repeated twice.

### 2.5 Effects of MT-DZY 6715 on the leaf structure of *C. oleifera*

The treatment for *C. oleifera* seedlings was conducted as described in section 2.4. Each seedling received 20 mL of the respective solution, which was applied evenly to ensure no droplets fell off. Leaf samples from the *C. oleifera* seedlings were collected 30 days post-treatment for further analysis.

#### 2.5.1 Scanning electron microscopy

The method for the scanning electron microscope was adapted from the previous description by Lubna et al. (2019) with appropriate modifications. Specifically, collected leaf samples were promptly immersed in 2.5% glutaraldehyde for overnight fixation, then rinsed with 0.1M phosphate-buffered saline (PBS) at a pH of 7.4, followed by dehydration with increasing concentrations of ethanol. After freeze-drying, the surfaces of the samples were coated with gold to enhance their visibility. Finally, the samples were observed under a Hitachi Regulus 8100 SEM.

#### 2.5.2 Stomata characteristics

The stomatal characteristics on the leaf surface were analyzed based on the method described by Ayala-Ramos et al. (2024), with slight modifications. Fully expanded and well-grown leaves were selected, and samples were collected between 8–9 am to ensure consistent physiological conditions. The lower epidermis of each leaf was meticulously peeled off using a fine dissecting knife, gently rinsed with sterile distilled water to remove any contaminants, and promptly transfer to phosphate-buffered saline (PBS) solution to maintain cell integrity. Stomatal characteristics, including the horizontal axis of stomata (HAS), vertical axis of stomata (VAS), stomatal area (SA), and stomatal perimeter (SP), were measured. For each observation, ten random fields of view were examined to ensure comprehensive assessment of the stomatal characteristics. Each treatment was repeated three times.

#### 2.5.3 Leaf tissue structure

The anatomical structure of the leaf was analyzed using the paraffin sectioning method (Yao et al., 2023). Specifically, collected samples were cut into 1x1 cm pieces using a dissecting knife and preserved in FAA solution (a mixture of formaldehyde, acetic acid, and 70% ethanol). Then, the samples were dehydrated in a series of ethanol solutions and ethanol/xylene mixtures, embedded in paraffin, and then sectioned using a Leica RM 2016 rotary microtome (Leica, Nussloch, Germany), the sections were stained with safranine O-fast green staining (Hu et al., 2022). Images of the stained sections were captured using a Zeiss MC 80 Axiolab optical microscope. The NDP view 2.9.22 RUO software was utilized to measure anatomical structures, including leaf thickness (Ln), the thickness of the upper (Tup) and lower epidermises (Tep), spongy mesophyll (Tsp), palisade mesophyll (Tpa), the ratio of palisade mesophyll to spongy mesophyll (PS). Additionally, tissue compactness (CTR) was calculated as the ratio of leaf thickness to palisade mesophyll thickness, and tissue looseness (SR) was assessed by the ratio of spongy mesophyll thickness to leaf thickness. Each treatment group consisted of three leaves, and the experiment was repeated twice.

### 2.6 Transcriptome analysis of *C. oleifera* induced by MT-DZY 6715

#### 2.6.1 RNA extraction, RNA-sequencing and differentially expressed gene identification

Samples of *C. oleifera* leaves, treated with MT, DZY 6715, and MT-DZY 6715 for 24 h, as well as a control group (CK) treated with sterile water, were collected and promptly frozen in liquid nitrogen for RNA extraction. The quality of the extracted RNA was evaluated using an Agilent 2100 Bioanalyzer. Subsequently, sequencing of the RNA libraries was conducted on the Illumina HiSeq platform. Low-quality reads were excluded from the raw sequencing data, resulting in a set of high-quality, filtered reads. These reads were then aligned to the reference genome of *C. oleifera* which was obtained from a designated website (https://zenodo.org/record/5768785#.ywrqgnzbwdu). Differentially expressed genes (DEGs) were identified using the DESeq2 software package. The functional annotation of these DEGs was conducted using the KEGG database (http://www.genome.jp/kegg/). To identify DEGs, we applied thresholds of |log2FC|>1 and a P <0.05.

#### 2.6.2 RNA-seq verification by quantitative real-time PCR

To verify DEGs expression, RT-qPCR was conducted, utilizing primers specifically designed for DEGs with Primer Express Software v2.0 (**Supplementary Table S1**), in accordance with the transcriptional analysis.

### 2.7 Effect of MT-DZY 6715 of on the defense enzymes activity and substances related to the cell wall in *C. oleifera*

The treatment method for *C. oleifera* remains consistent with that described in section 2.4. Leaf samples of *C. oleifera*, collected on the 30th day post-treatment, were used to evaluate the following indicators. The activity of phenylalanine ammonia-lyase (PAL) was assessed as described by Zhu et al. (2021). The activities of cinnamic acid-4-hydroxylase (C4H) and 4-coumarate: coenzyme A ligase (4CL) were investigated according to the method described by Yin et al. (2023). Laccase and cinnamyl-alcohol dehydrogenase (CAD) activities were measured in accordance with the protocol established by Khedr and Khedr (2024). The content of lignin and cellulose were examined as illustrated by Sun et al. (2023). Additionally, cellulase activities was examined utilizing the methodology detailed by Abu-Goukh and Bashir (2003). Protopectin content was determined based on Shinga and Fawole (2023) method. Pectinase activity was determined as described by Dal Magro et al. (2019). The results for the activities of PAL, 4CL, C4H, CAD, laccase, cellulase, and pectinase are expressed in units per gram of protein concentration (U g^-^¹), while the content of lignin, cellulose, and protopectin is indicated as milligrams per gram of fresh leaf weight (mg g^-^¹).

### 2.8 Statistical analyses

Statistical comparisons of the data were performed using independent *t-test* or Duncan’s multiple range test in SPSS 20.0 (IBM, Armonk, NY, USA), with statistical significance determined at *P <*0.05.

## 3 Result

### 3.1 Inhibitory effect of MT-DZY 6715 against *C. siamense in vitro*

**Figure 1:**
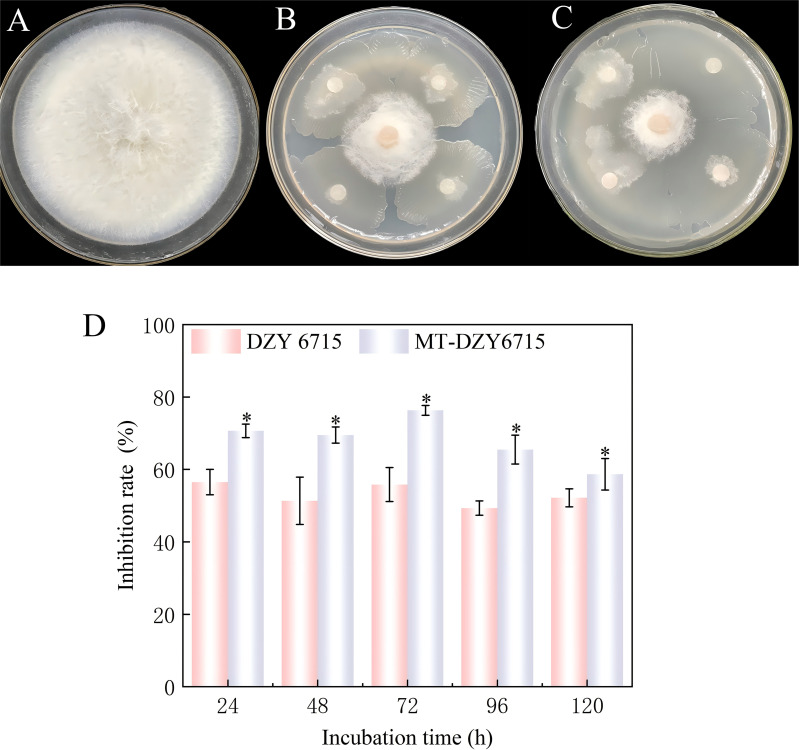
Representative photos demonstrating the antagonistic activity of MT-DZY 6715 on the growth of *C. siamense.* **(A)** represents the treatment with the pathogen alone; **(B)** shows the treatment with DZY 6715 suspension; **(C)** displays the treatment with MT-DZY 6715; and **(D)** illustrates the inhibitory effect on the pathogen *in vitro*. The data presented are expressed as the mean ± standard error (SE). * represents significant differences based on *P <*0.05.

After inducing DZY 6715 with 50 μmol L^-1^ MT (MT-DZY 6715), the inhibitory effect on *C. siamense* was the best, during the 24–120 h of cultivation, MT-DZY6715 treatment showed an inhibition rate of 58.67% -76.33% against *C. siamense*, and the highest inhibition rate was observed at 72 h, which was 26.86% higher than the DZY 6715 treatment (**Figure 1**).

### 3.2 The impact of MT-DZY 6715 aganist *C. siamense* on *C. oleifera*

**Figure 2:**
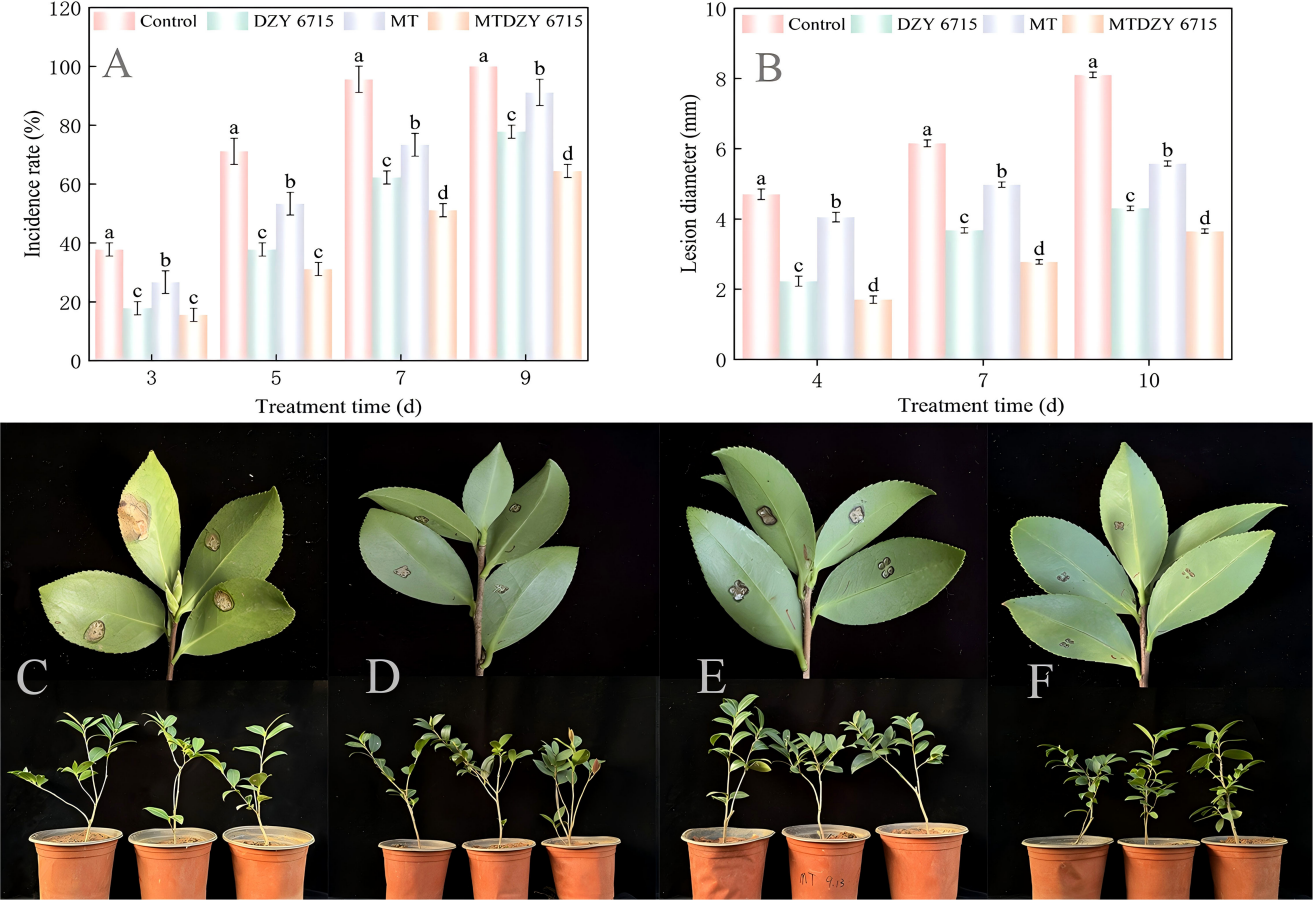
The incidence rate **(A)** and lesion diameter **(B)** of *C. siamense* on *C. oleifera* leaves during the cultivation period. Representative images illustrate the effect of MT-DZY 6715 on lesion development in *C. oleifera* leaves resulting from 10 days post-pathogen inoculation **(C-F)**. **(C-F)** represent treatments with *C. siamense*, DZY 6715, MT, and MT-DZY 6715, respectively. Data are represented as mean ± standard error (SE). Diverse letters are used to denote significantly differences among the different timepoints as determined through a Duncan’s multiple range test (*P < 0.05*).

**Figure 2A** showed that as the treatment time increased, the disease incidence rate in all treatment groups rises linearly. At the 9th day, the disease incidence rate in the control group reached 100%, which is significantly higher than that of the DZY 6715, MT, and MT-DZY 6715 treatment groups by 22.22%, 8.89%, and 35.56%, respectively. In addition, the lesion diameter on the leaves of each treatment group also gradually increase with prolonged treatment time. However, from the 4th to 10th day of treatment, the leaf lesion diameters of the DZY 6715, MT, and MT-DZY 6715 treatment are significantly lower by 40.24-52.66%, 13.83-31.17%, and 54.88-63.83%, respectively, compared to the control group (**Figure 2B**). The above indicated that the MT-DZY 6715 treatment exhibited the highest inhibitory activity against *C. oleifera* anthracnose caused by *C. siamense*.

### 3.3 Impact of MT DZY 6715 on the leaf structure of *C. oleifera*

#### 3.3.1 Surface structure and stomata of *C. oleifera* leaves

**Figure 3:**
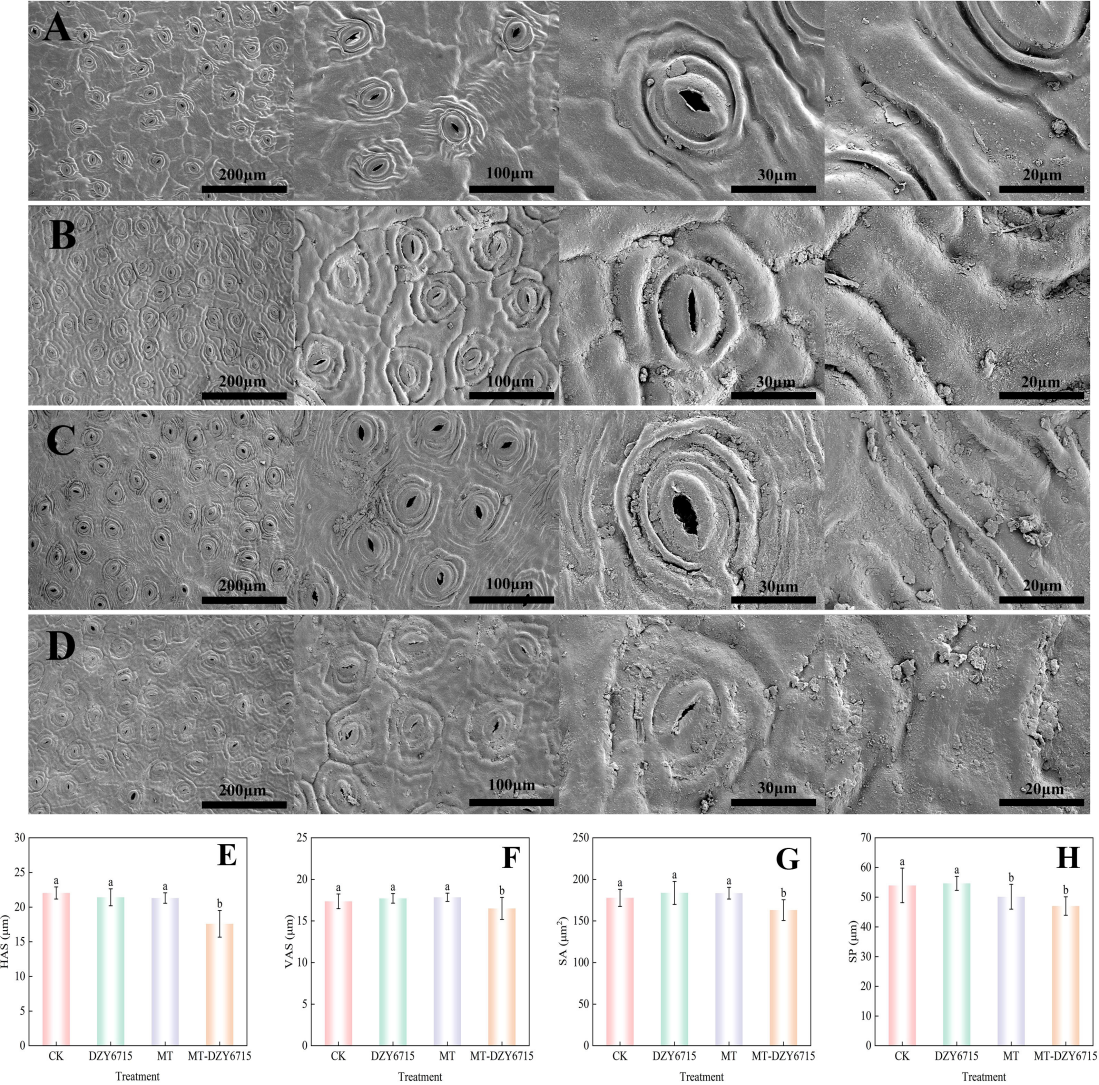
Scanning electron microscope assessment of the effects of CK **(A)**, DZY 6715 **(B)**, MT **(C)**, and MT-DZY 6715 **(D)** treatments on the stomata of *C. oleifera* leaves, and stomatal structure characteristic parameters of leaves under different treatments at 30 d: HAS **(E)**, VAS **(F)**, SA **(G)**, and SP **(H)**. Data are represented as mean ± standard error (SE). Different letters indicate significant differences between the different treatments as determined by a Duncan’s multiple range test (*P < 0.05*).

**Figure 3** showed that on the 30th day post-treatment, the MT-DZY 6715 treatment group exhibited stomatal dimensions with horizontal axis of stomata (HAS) of 17.59 μm, a vertical axis of stomata (VAS) of 16.51 μm, a stomatal area (SA) of 163.12 μm^2^, and a stomatal perimeter (SP) of 47.03 μm. These values were significantly (*P*<0.05) lower compared to the CK, MT, and DZY 6175 treatment groups. Furthermore, the stomata in the MT-DZY 6715 treated group were nearly fully closed, meanwhile, the stomata in the control and MT groups were larger and more open. In addition, after 30 days of treatment, the epidermal surfaces of *C. oleifera* leaves showed varying amounts of solid particulate matter. It is worth noting that compared with the control group, DZY 6715, and MT, the leaves treated with MT-DZY 6715 showed notably more solid particles. These particles comprised the waxy layer of the leaf epidermis. Furthermore, MT-DZY 6715 treatment resulted in reduced epidermal folding and a wax layer with dense, fragmented protrusions.

#### 3.3.2 Leaf anatomical structure

**Figure 4:**
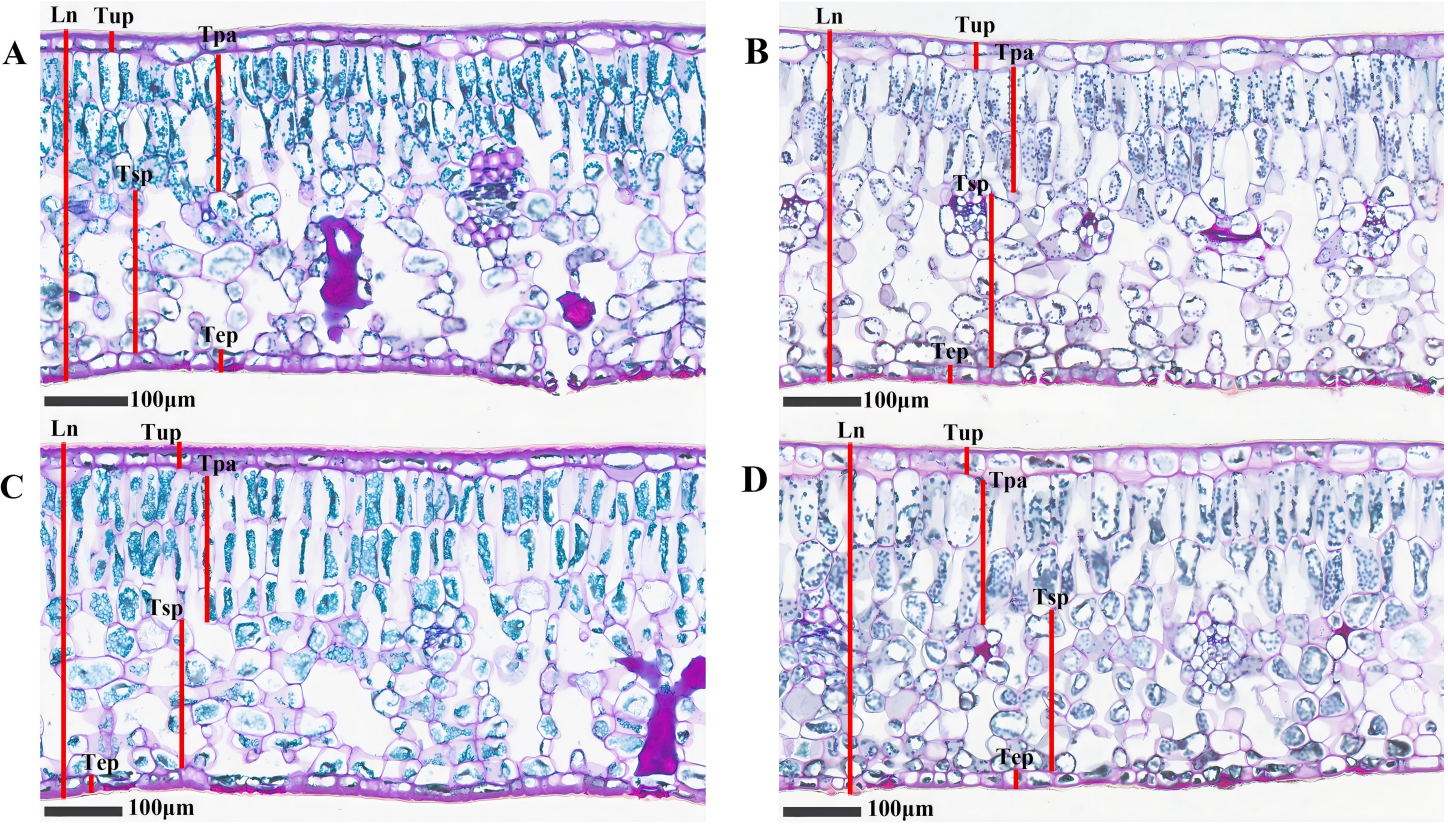
Microscopic analysis of *C. oleifera* leaf anatomical structures at ×20 magnification after treated with CK **(A)**, DZY 6715 **(B)**, MT **(C)**, MT-DZY 6715 **(D)**.

**Figure 5:**
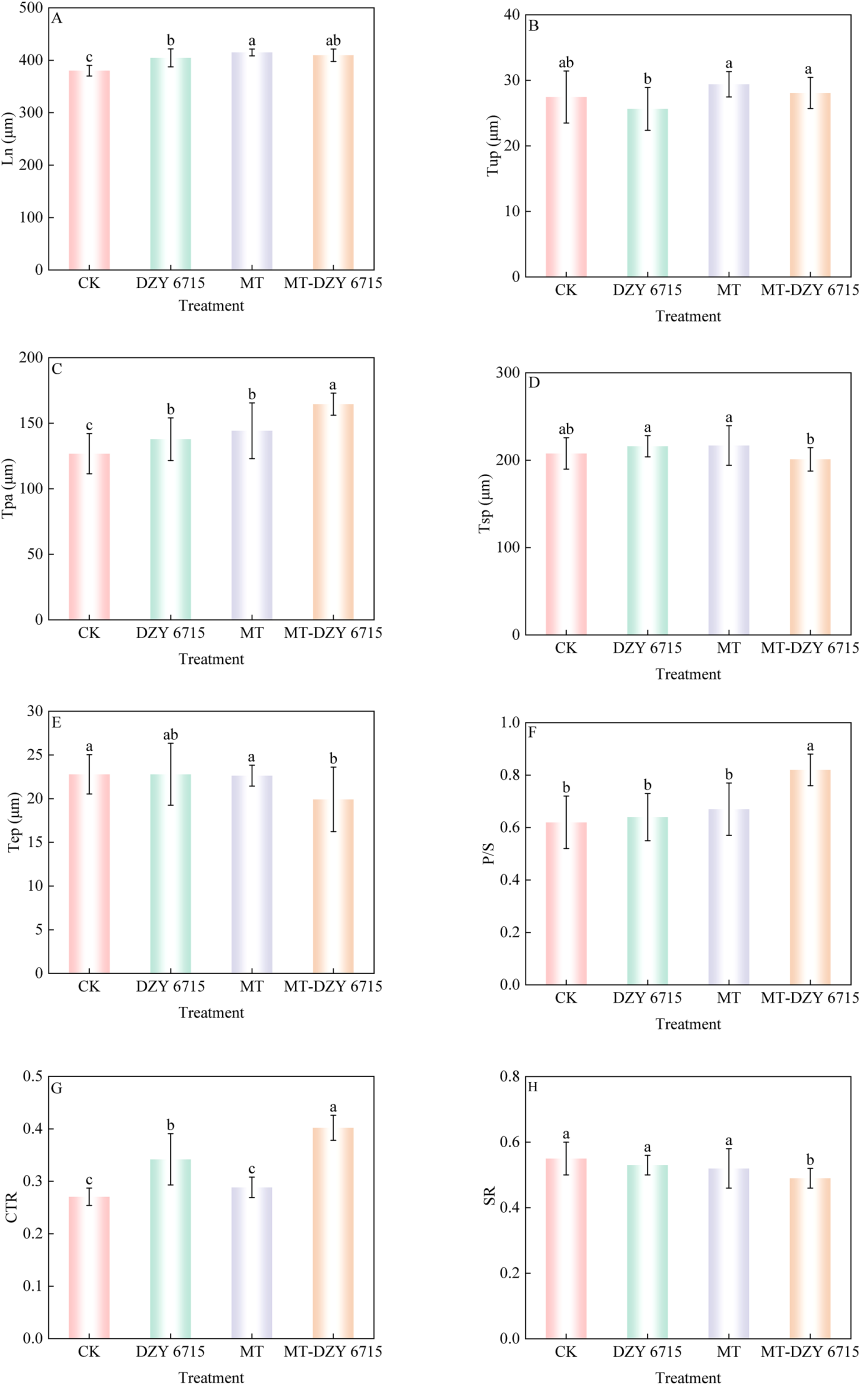
Morpho-anatomical traits of *C. oleifera* leaf of different treatments at 30(d) **(A)** Ln, leaf thickness; **(B)** Tup, thickness of the upper epidermises; **(C)** Tpa, thickness of the palisade mesophyll; **(D)** Tsp, thickness of the spongy mesophyll; **(E)** Tep, thickness of the lower epidermises; **(F)** P/S: the ratio of palisade mesophyll to spongy mesophyll; **(G)** CTR (tissue compactness), the ratio of leaf thickness to palisade mesophyll thickness; **(H)** SR (tissue looseness), the ratio of spongy mesophyll thickness to leaf thickness. Vertical bars indicate the standard errors of the mean. Different letters denote a significant difference among various treatments, as determined by one-way ANOVA at *P* < 0.05. Values are the mean ± SE.

**Figure 4** exhibited the structural features of the cross-section of *C. oleifera* leaves. Both the upper and lower epidermal layers are composed of a single layer cell. Adjacent to the upper epidermis is the palisade mesophyll, characterized by its tightly packed arrangement of elongated, columnar cells in 2–3 layers. Spongy mesophyll, situated next to the palisade mesophyll, consists of irregularly shaped cells arranged loosely with expansive intercellular spaces. **Figure 5** showed that there are differences in the anatomical characteristics of *C. oleifera* leaves following various treatments. Compared to the control, leaves treated with DZY 6715, MT, and MT-DZY 6715 exhibited an increase in leaf thickness (Ln) and palisade mesophyll thickness (Tpa). No significant difference was observed in the upper/lower epidermal thickness (Tup/Tep) between the MT-DZY 6715 treatment and the control. The sponge tissue thickness (Tsp) in the MT-DZY6715 treatment group was significantly reduced compared to the control, MT and DZY 6715 treatment groups. Additionally, leaves treated with MT-DZY 6715 exhibited a significantly higher ratio of palisade mesophyll to spongy mesophyll (PS) and a greater degree tissue compactness (CTR) than the control, MT, and DZY 6715 groups. Conversely, the tissue looseness (SR) in the MT-DZY6715 treatment group was significantly lower than that in the other three groups.

### 3.4 Transcriptomic analysis of *C. oleifera* treated with MT-DZY 6715

#### 3.4.1 DEGs identified by transcriptome analysis

**Figure 6:**
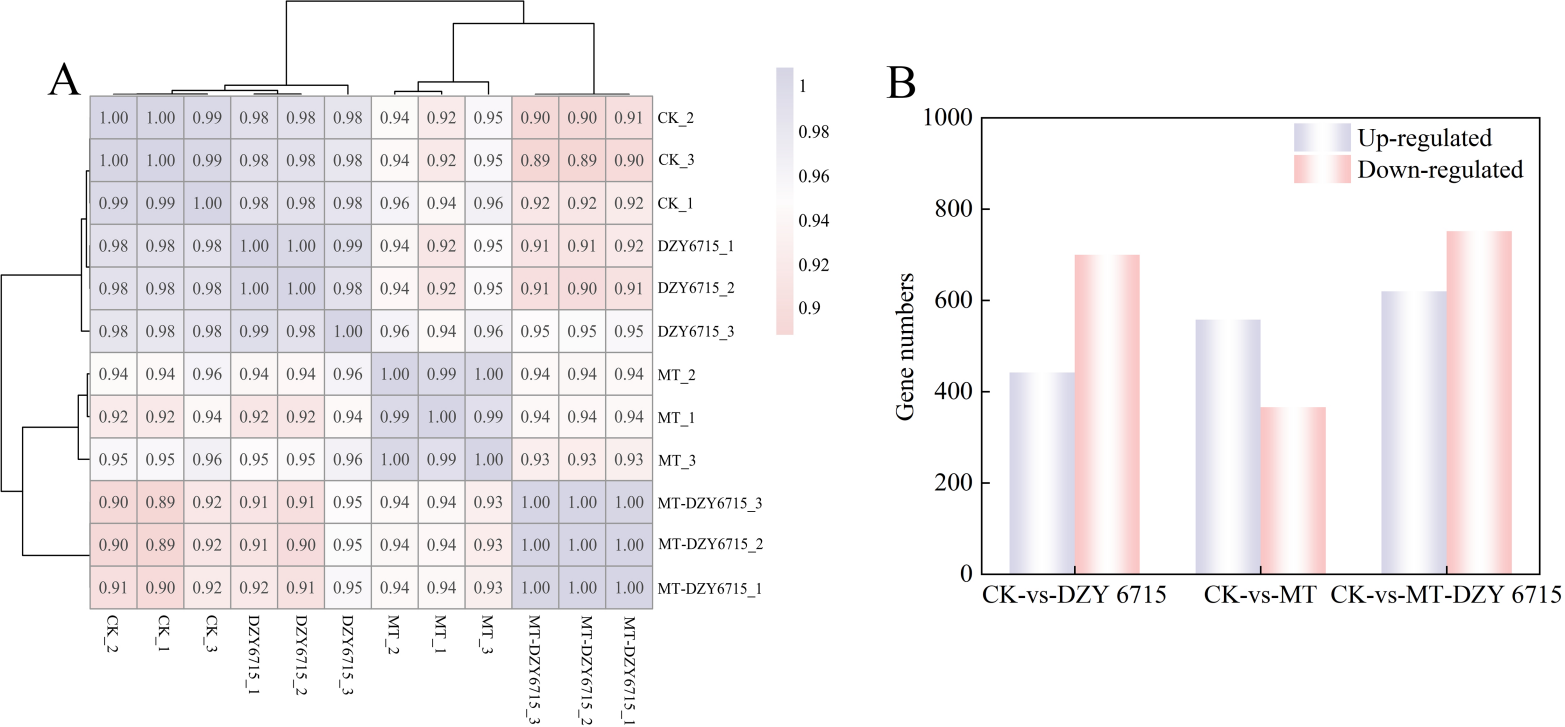
Sample correlation test chart **(A)**, and DEGs in CK-vs-DZY 6715, CK-vs-MT, and CK-vs-MT-DZY 6715 **(B)**.

In the present study, the R^2^ values between biological replicates fell within the range of 0.98 to 1.00, signifying the high reliability of the transcriptome data, thereby validating it for subsequent downstream analyses (**Figure 6A**). In the comparison CK-vs-DZY 6715, 1,142 differentially expressed genes (DEGs) were detected, with 442 upregulated and 700 downregulated. In CK-vs-MT, 924 DEGs were identified, including 558 upregulated and 366 downregulated genes. A total of 1,372 DEGs were detected in CK-vs-MT-DZY 6715, among which 620 were upregulated and 752 were downregulated. The identification of a higher number of DEGs in CK-vs-MT-DZY 6715 suggests that the MTDZY 6715 treatment has a more significant impact on *C. oleifera* (**Figure 6B**).

#### 3.4.2 Effects of different treatments on the transcriptome of *C. oleifera*

**Figure 7:**
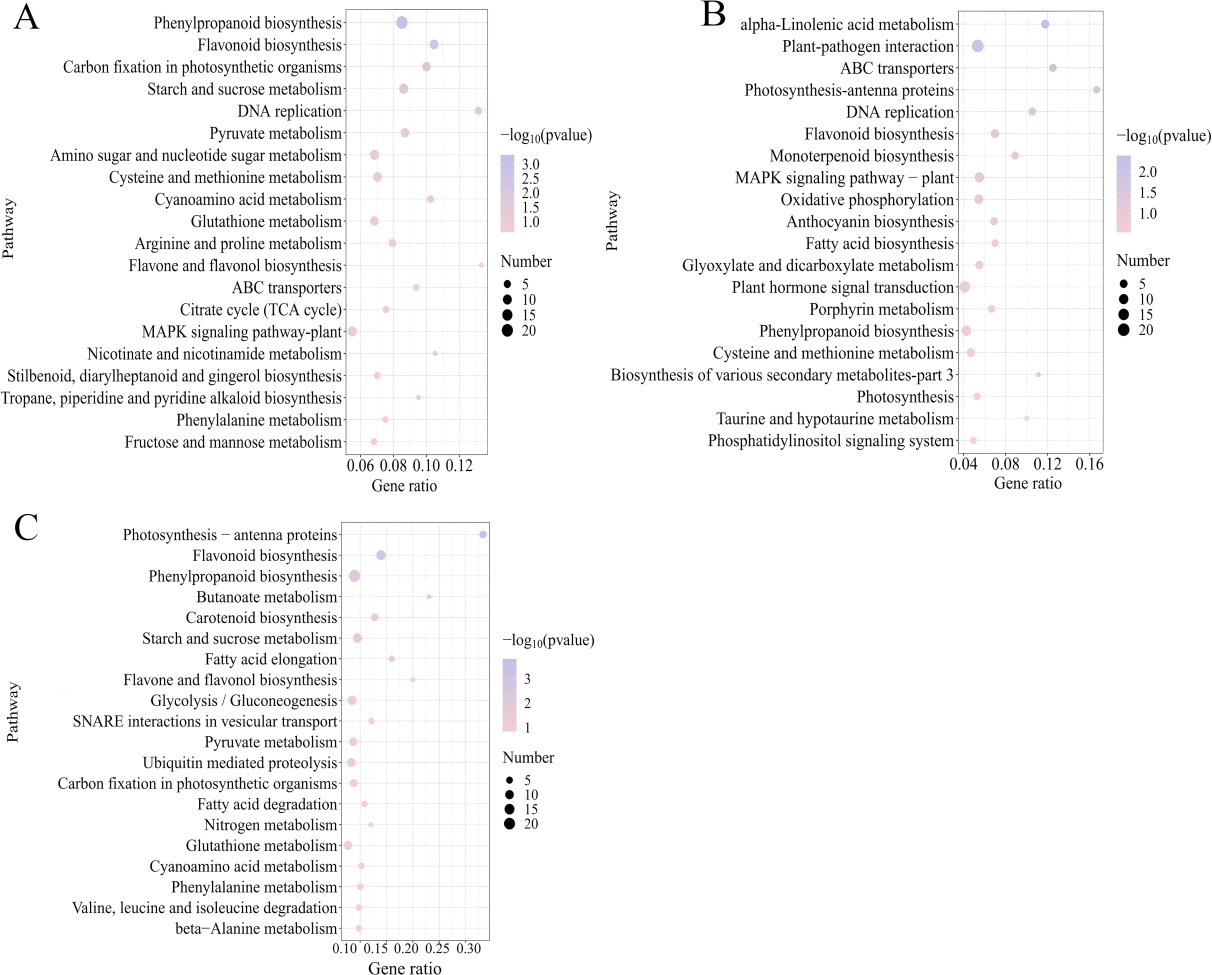
The 20 most significantly associated KEGG pathways identified in comparisons of CK-vs-DZY 6715 **(A)**, CK-vs-MT **(B)**, CK-vs-MT-DZY 6715 **(C)** in *C. oleifera*.

To further elucidate gene functions, KEGG enrichment analysis was conducted on the DEGs. The results revealed that the 1,142, 924, and 1,372 DEGs identified in CK-vs-DZY 6715, CK-vs-MT, and CK-vs-MTDZY 6715, respectively, were enriched into 92, 85, and 108 KEGG pathways, with the top 20 pathways shown in the **Figure 7**. Among these top 20 KEGG pathways, Phenylpropanoid biosynthesis exhibited the highest number of enrichments in both CK-vs-DZY 6715 (**Figure 7A**) and CK-vs-MT-DZY 6715 (**Figure 7B**), whereas Plant-pathogen interaction was the most enriched in CK-vs-MT (**Figure 7C**). The application of DZY 6715, MT, and MT-DZY 6715 altered the differential gene expression (DEGs) link to disease resistance in *C. oleifera*. These DEGs were subsequently analyzed and categorized into four functional groups: transcription factors, plant hormone signal transduction, cell wall and phenylpropanoid metabolism.

**Figure 8:**
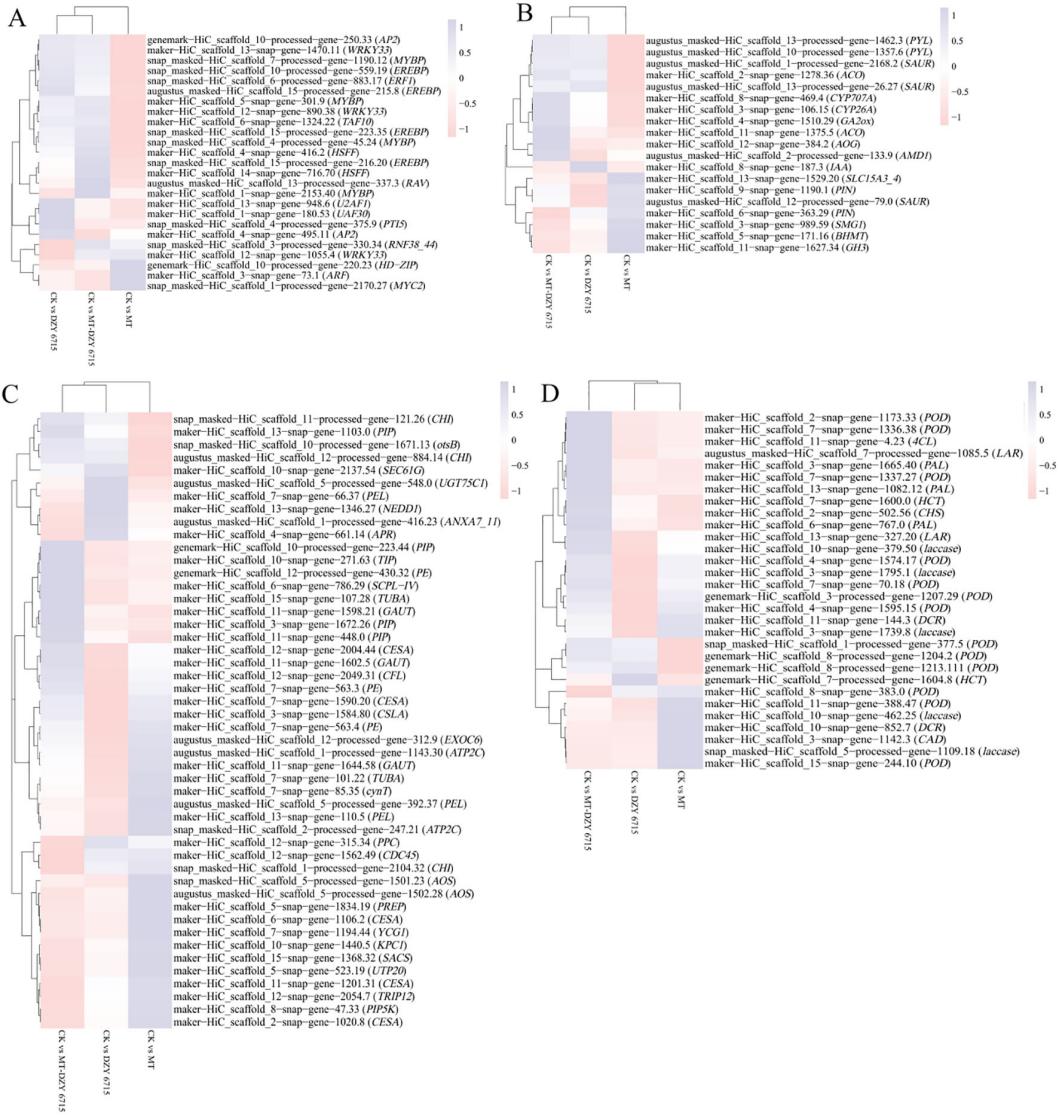
presents cluster analyses of the expression profiles of differentially expressed genes (DEGs) related to transcription factors **(A)**, plant hormone signal transduction **(B)**, cell wall metabolism **(C)**, and phenylpropanoid metabolism **(D)** following treatment with DZY 6715, MT, MT-DZY6715.

For transcription factors (**Figure 8A**), compared to the control group, after DZY 6715 application, a total of 13 genes were up-regulated, including *TAF10*, *MYBP*, *U2AF1*, *ERF1*, *PTI5*, *UAF30*, *2 AP2*, *3 EREBPs*, and *2 WRKY33*, while 5 genes were down-regulated, encompassing *HD-ZIP*, *HSPP*, *WRKY33*, *2 MYBPs*, and *ARF*. Following the MT application, 6 genes experienced up-regulated, including *AP2*, *MYC2*, *WRKY33*, *ARF*, *UAF30* and *RNF38_44*, while 7 genes were down-regulated, including *EREBP*, *HD-ZIP*, *HSFF*, *WRKY33*, and *3 MYBPs*. The application of MT-DZY 6715, 16 genes exhibited up-regulation, such as *TAF10*, *AP2*, *U2AF1*, *RAV*, *ERF1*, *HSFF*, *UAF30*, *RNF38_44*, along with *3 EREBPs*, *3 MYBP* and *2 WRKY33*, while 5 genes were down-regulated, including *HD-ZIP*, *WRKY33*, *ARF*. For plant hormone signal transduction (**Figure 8B**), compared to the control group, after DZY 6715 was applied, 11 genes were affected, with 3 up-regulated (including *AOC*, *IAA*, *SAUR*), and 8 genes down-regulated (*BHMT*, *SMG1*, *AMD1*, *SAUR*, *PIN*, *GH3*, *PYL*, and *SLC15A3_4*). And the MT application, 9 genes exhibited changes, with 4 up-regulated, such as *IAA*, *GH3*, *SAUR*, *AUX1*, and 5 down-regulated, including *BHMT*, *CYP26A*, *SAUR* and *2 PYL*. Meanwhile, MT-DZY6715 treatment led to alterations in 13 genes. Among these, 7 genes were up-regulated, including *GA2ox*, *CYP707A*, *IAA*, *SAUR*, *AOG* and *2 AOCs*, while 6 genes down-regulated like *BHMT*, *SMG1*, *PIN*, *GH3*, *PYL*, *SLC15A3_4*. For cell wall metabolism (**Figure 8C**), compared to the control group, after DZY 6715 was applied, 29 genes were affected, among these, 6 genes were up-regulated, including *otsB*, *SEC61G*, *PIP*, *ANXA7_11* and *2 CHI*. At the same time, 23 genes were down-regulated, such as *PE*, *CHI*, *PREP*, *2 ATP2C*, *PPC*, *3 PEL*, *CFL*, *CDC45*, *YCG1*, *TUBA*, *3 CESA*, *2 GAUT*, *CSLA*, *SCPL-IV*, *NEDD1*, *SACS*, *EXOC6*. Following MT application, 18 genes exhibited changes, with 8 up-regulated (*PIP5K*, *PE*, cynT, *2 AOS*, *PEL*, *TUBA*, *CESA*) and 10 down-regulated (*PPC*, *PEL*, *CFL*, *CDC45*, *UGT75C1*, *GAUT*, *NEDD1*, *ECOC6* and *2 CHI*). Meanwhile, MT-DZY6715 treatment led to alterations in 34 genes. Among these, 13 genes were up-regulated, such as *PE*, *otsB*, *2 CHI*, *TUBA*, *4 PIP*, *TIP*, *CESA*, *GAUT*, *SCPL-IV*. While 21 genes were down-regulated, including *PIP5K*, *CHI*, *PREP*, *ATP2C*, *PPC*, *3 PEL*, *APR*, *CDC45*, *YCG1*, *TUBA*, *TRIP12*, *2 CESA*, *KPC1*, *UGT75C1*, *UTP20*, *NEDD1*, *SACS*, *EXOC6*. In the phenylpropanoid metabolism (**Figure 8D**), the application of DZY6715 resulted in the differential expression of 22 genes compared to the control group. Specifically, 8 genes were up-regulated, including 6 peroxidases (*PODs*) and 2 hydroxycinnamoyl-CoA shikimate/quinate hydroxycinnamoyltransferases (*HCTs*), while 14 genes were down-regulated, comprising 5 *PODs*, 5 laccases (*LAs*), 2 leucoanthocyanidin reductases (*LARs*), and 2 cinnamoyl-CoA reductases (*DCRs*). Upon the application of MT, 14 lignin-associated genes exhibited altered expression, with 9 genes up-regulated, including 7 *PODs* and 2 *HCTs*, and 5 genes down-regulated, specifically 2 *PODs*, 2 *LARs*, and chalcone synthase (*CHS*). The application of MT-DZY6715 led to changes in the expression of 15 phenylpropane metabolism-related genes, with 15 genes up-regulated, including 7 *PODs*, CHS, 4-coumarate-CoA ligase (*4CL*), LA, 3 phenylalanine ammonia-lyases (*PALs*), and 2 *HCTs*, while 5 genes were down-regulated, including *POD*, 3 *LAs*, and *DCR*.

#### 3.4.3 qRT-PCR assay

**Figure 9:**
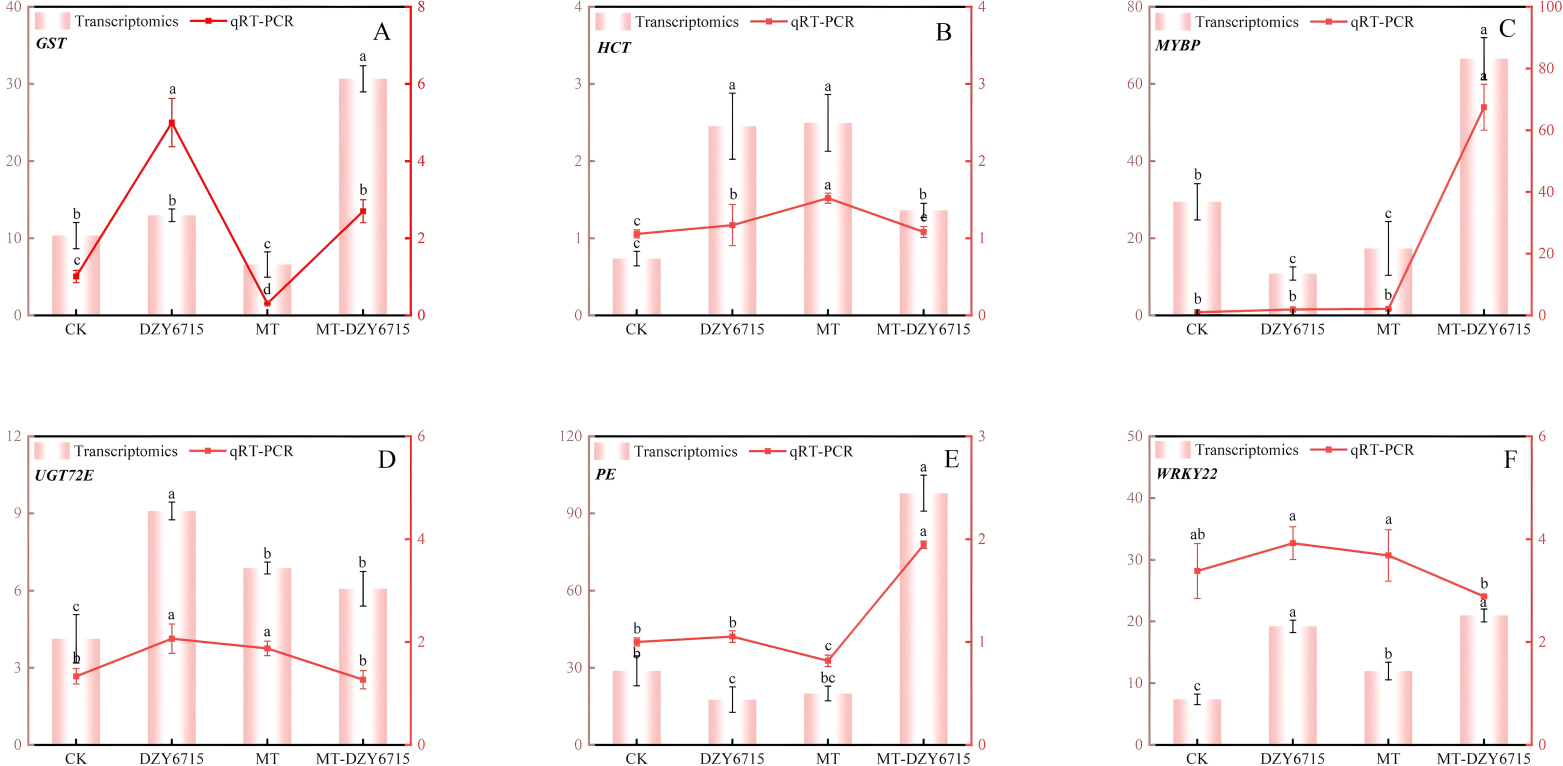
qRT-PCR assay performed to validate the DEGs *GST* **(A)**, *HCT* **(B)**, *MYBP* **(C)**, *UGT72E* **(D)**, *PE* **(E)**, *WRKY22* **(F)**. The vertical bars indicate the SD from three replicates. Different letters indicate significant differences among the different treatment groups, as determined by Duncan’s multiple range test (*P < 0.05*).

To verify the accuracy of the transcriptome data, we performed qRT-PCR analysis on six randomly selected differentially expressed genes. The results, as illustrated in **Figure 9**, demonstrate that the expression levels of these genes in the CK, DZY 6715, MT, and MT-DZY 6715 samples align with the trends observed in the transcriptome data, indicating the reproducibility and consistency of the RNA-seq data.

### 3.5 Defense enzyme activities and substances in *C. oleifera* after MT-DZY 6715 treatment

**Figure 10:**
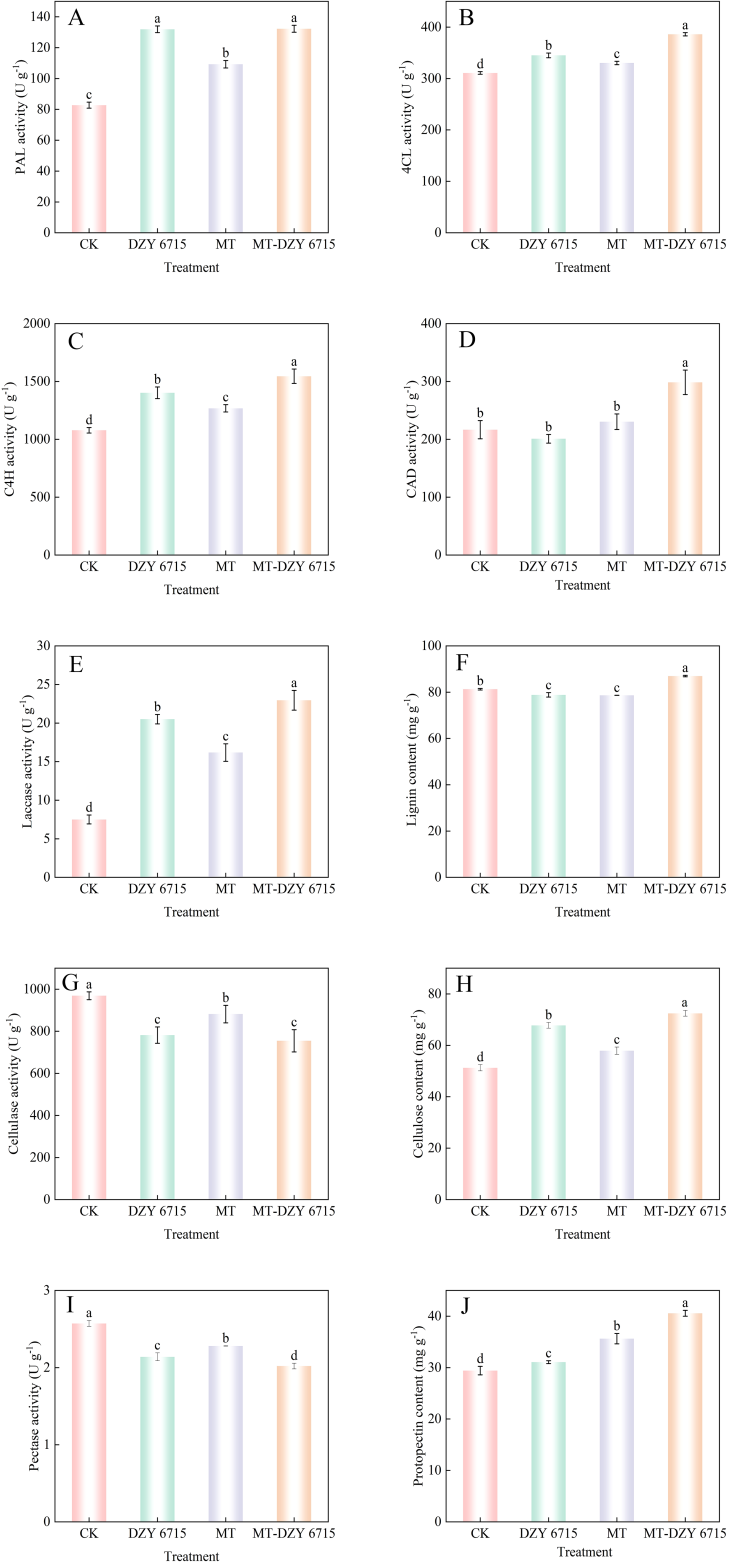
Effect of MT-DZY 6715 on the PAL **(A)**, 4CL **(B)**, C4H **(C)**, CAD **(D)**, laccase **(E)** cellulase **(G)**, pectinase **(I)** activities and the content of lignin **(F)**, cellulose **(H)**, protopectin **(J)** in *C. oleifera*. Diverse letters stand for significantly different at the different treatment groups as determined through a Duncan’s multiple range test (*P < 0.05*).

After 30 days of treatment with DZY 6715, MT, and MT-DZY6715, the PAL activity in *C. oleifera* significantly increased by 59.50%, 32.11%, and 59.86%, respectively, compared to the control group (**Figure 10A**). Similarly, the 4CL activity in the groups treated with DZY 6715, MT, and MT-DZY6715 was significantly elevated compared to the control. Specifically, the 4CL activity was 344.97 U g^-1^ (10.99% higher), 330.29 U g^-1^ (5.65% higher), and 386.0 U g^-1^ (24.20% higher) in the DZY 6715, MT, and MT-DZY 6715 groups, respectively (**Figure 10B**). Additionally, after 30 days of treatment with DZY 6715, MT, and MT-DZY 6715, the C4H activity significantly rose by 30.04%, 17.60%, and 43.28%, respectively, compared to the control group (**Figure 10C**). On the 30th day of treatment, the CAD activity in the DZY 6715-treated group was reduced to 1.08- times that of the control group, whereas the CAD activity in the MT and MT-DZY 6715 treatment groups was elevated to 1.06- times and 1.38- times that of the control group, respectively (**Figure 10D**). As shown in **Figure 10E**, the laccase activity in the treatment groups with DZY 6715, MT, and MT-DZY 6715 was 2.73-, 2.16-, and 3.06- times higher, respectively, compared to that of the control group. **Figure 10F** exhibited that after 30 days of treatment, the lignin content in the DZY 6715 and MT treatment groups was 3.02% and 3.22% lower, respectively, compared to the control group. In contrast, the lignin content in the MT-DZY6715 treatment group was 6.96% higher than that of the control group. On the 30th day, the cellulase activities in the DZY 6715, MT, and MT-DZY6715 treatment groups were 781.88, 881.96, and 754.85 U g^-1^, respectively, which were 23.94%, 9.88%, and 28.38% lower than those in the control (**Figure 10G**). As shown in **Figure 10H**, the cellulose content in the DZY 6715, MT, and MT-DZY 6715 treatment groups significantly increased to 1.32- times, 1.13- times, and 1.41- times higher than that of the control group, respectively. On the 30th day of treatment, the pectinase activity in the control group was 1.20-, 1.13-, and 1.27- times higher than that in the DZY 6715, MT, and MT-DZY 6715 treatment groups, respectively (**Figure 10I**). **Figure 10J** showed that on the 30th day, the protopectin content in the DZY 6715, MT, and MT-DZY6715 treatment groups was markedly higher than that in the control group, with increases of 5.60%, 21.14%, and 37.92%, respectively.

## 4 Discussion

Anthracnose, a disease primarily caused by *Colletotrichum* species like *C. gloeosporioides*, *C. fructicola*, *C. siamense*, and *C. boninense*, poses as a significant threat to *C. oleifera* (Jeyaraj et al., 2023). This disease results in substantial yield losses, ranging between 20% and 50% (Zhu and He, 2023). In response to the growing emphasis on environmental protection, the application of *Bacillus* species for microbial control has emerged as a more sustainable and efficacious alternative (Elnahal et al., 2022; Lastochkina et al., 2019). Research has shown that *Bacillus* species effectively inhibit the growth and invasion of pathogen through a combination of mechanisms, including nutrients spatial niche competition (Caulier et al., 2018; Lastochkina et al., 2019), secretion of antibiotic substances, production of antibacterial proteins (Liu et al., 2020; Zhang et al., 2017), and induction of enhanced activity of plant disease resistance-related enzymes (Bai et al., 2023; Fan et al., 2023; Wu et al., 2019). Although *Bacillus* sp. exhibit considerable potential for biological control, they are generally less effective than chemical fungicides. MT, as a naturally occurring small molecule substance widely present in organisms, functions as a biological stimulant capable of counteracting various biotic and abiotic stresses (Ahammed et al., 2020; Arnao and Hernández-Ruiz, 2014; Zhao et al., 2019). As reported by Liu et al. (2024), the combined application of MT and fungal metabolites enhances disease resistance in tomato plants against bacterial wilt by regulating the expression of plant hormone-related genes, increasing the contents of jasmonic acid (JA) and salicylic acid (SA), decreasing ethylene (ET) levels, and augmenting antioxidant enzyme activity within the plants. Chen et al. (2019) indicated that compared to the control, MT significantly inhibited the growth of *Xanthomonas oryzae* pv. *oryzicola* (Xoc), reduced its pathogenicity, as evidenced by a decrease in lesion length by over 23% and a reduction in bacterial population by 45%. Additionally, MT effectively suppressed Xoc’s biofilm formation, motility, and extracellular protease activity, ultimately leading to a 17% decrease in the incidence of rice bacterial leaf streak disease. In the present work, MT was used as the elicitor for the induced culture of *B.tequilensis* DZY 6715. According to our results, MT-DZY 6715 exhibited enhanced antibacterial activity compared to DZY 6715, achieving a maximum inhibition rate of 76% against *C. siamense in vitro*. Furthermore, MT-DZY 6715 treatment effectively suppressed the growth of the anthracnose pathogen and controlled its spread on leaves of *C. oleifera*.

Leaves, being highly sensitive to environmental changes and possessing significant plasticity, provide an important perspective through the study of their anatomical structures for analyzing the physical defense mechanisms and disease resistance against pathogens (Accioly et al., 2024). Specifically, leaf epidermis serves as the first barrier between plants and the external environment. It often contains structures such as wax layer and cuticle layer, which are crucial in resisting pathogen invasion (Wang et al., 2020b; Ziv et al., 2018). The wax layer, as a protective barrier outside the cuticle, endows plants with natural defense capabilities, as its hydrophobic properties can effectively prevent pathogen spores from adhering to the leaf surface, thus hindering their penetration into plant tissues (Li et al., 2019; Reina-Pinto and Yephremov, 2009; Sharma et al., 2018; Tafolla-Arellano et al., 2018). *C. oleifera* anthracnose belongs to an air-borne disease, with the pathogen producing a large number of conidia that spreads through media like wind and rain. In the current research, compared to the control, the epidermal wax of leaves treated with MT-DZY 6715 showed dense, fragmented, and more tightly packed protrusions. These changes indicated that the MT-DZY 6715 treatment altered the structure of the wax crystals on the *C. oleifera* leaves, forming a stronger defensive barrier, and thus more effectively resisting pathogen invasion. In addition, thicker leaves provide plants with a more solid physical barrier, which not only effectively blocks pathogen entry into plant tissues, mitigating disease incidence, and aiding in water conservation by reducing transpiration, thereby maintaining the plant’s water balance (Guo et al., 2017; Ma et al., 2012). As the same time, thicker leaves exhibit heightened efficiency in utilizing light energy to synthesize organic matter (Jinwen et al., 2009; Murchie et al., 2005), which helps accumulate more nutrients and enhance the plant’s resistance to diseases. The palisade mesophyll and spongy mesophyll are crucial mesophyll tissues. Palisade mesophyll is primarily responsible for efficiently conducting photosynthesis, thereby accumulating energy and nutrients for the plant (Zhang et al., 2022). Meanwhile, the spongy mesophyll features large cell gaps and primarily functions to store water and facilitate gas exchange, maintaining water balance and gas circulation within the plant (Tamang et al., 2023). Their synergistic effect is vital in maintaining leaf stability and enhancing plant’s disease resistance. For example, Wang et al. (2022b) found that the decreased expression of *CmNF-YB8* leads to improved drought resistance in *chrysanthemum* by altering leaf anatomy. Specifically, this modification includes a decrease in stomatal opening and the development of a thicker epidermal cuticle. Walnuts exhibit enhanced disease resistance against *Xanthomonas arboricola* pv. *juglandis* through altered leaf anatomy, characterized by reduced stomatal length and area, as well as an increased thickness ratio of spongy to palisade mesophyll (Yang et al., 2021). In our study, leaves treated with MT-DZY 6715 exhibited increased leaf thickness (Ln), palisade mesophyll thickness (Tpa) compared to the control group, and the treated leaves had a significantly higher ratio of palisade mesophyll to sponge tissue (PS) and tissue compactness (CTR), as well as reducing tissue looseness (SR) (**Figure 5**). Furthermore, the stomata of the leaves treated with MT-DZY6715 decrease in stomatal opening, and the stomatal characteristic index was also significantly lower compared to the control, MT, and DZY 6715 treatment (**Figure 3**). These structural changes further suggested that *C. oleifera* leaves treated with MT-DZY 6715 enhanced resistance to the infiltration of pathogens.

To further investigate the mechanism by which MT-DZY 6715 treatment enhances the disease resistance of *C. oleifera*, we analysis the molecular mechanisms underlying MT-DZY 6715-induced resistance enhancement using transcriptome. In this study, KEGG pathway enrichment analysis indicated that the secondary metabolites biosynthesis played a pivotal role in *C. oleifera* when treated with MT-DZY 6715. Previous studies have demonstrated that the synthesis of secondary metabolites in plants is significantly influenced by a range of transcription factors (TFs), including *WRKY* (Chen et al., 2017), *MYB* (Zhao et al., 2022b), *AP2/ERF* (Mizoi et al., 2012), *NAC* (Danielsson et al., 2011), *bHLH* (Sun et al., 2018). Among these, *WRKY* transcription factors play a pivotal role in regulating the biosynthesis of various secondary metabolites, like phenols, lignin, flavonoids, tannins, etc. For instance, Tang et al. (2023) research revealed that *Paeonia lactiflora* can regulate its secondary cell wall thickness to enhance stem strength through modulation by TF *PlWRKY41a*. Wang et al. (2022a) have demonstrated that group IIc *WRKY* TFs directly binds to the promoter of *GhMKK2*, regulating its expression and triggering a novel mitogen-activated protein kinase (MAPK) cascade involving *GhMKK2*, *GhNTF6*, and *GhMYC2*. By upregulating various flavonoid biosynthesis-related genes expressed by *GhMYC2*, the accumulation of flavonoids was increased, thereby enhancing cotton’s resistance to *Fusarium oxysporum* f. sp. *vasinfectum*. Similarly, *HvWRKY23* regulates the expression of genes such as *PAL*, *C4H*, *4CL*, *HCT*, *Laccase15*, and *UDPGT*, promoting the biosynthesis of favonoid glycoside and Hydroxycinnamic acid amides, which strengthens cell walls and enhances barley resistance to *Fusarium* head blight (Karre et al., 2019). In potatoes, *StNAC43* activates the *StMYB8*, and subsequently, *StMYB8* directly regulates the expression of secondary cell wall biosynthesis genes such as *HCT*, *PHT*, and *CHS*, enhancing the structure and function of cell wall, thereby increasing potato resistance to late blight (Yogendra et al., 2017). In addition, *GbERF1*-like, acting as a positive regulator of lignin synthesis, enhances lignin accumulation in cotton plants by promoting the expression of genes-related to plant secondary metabolism, including *PAL*, *C4H*, *C3H*, *HCT*, *CCR*, and *F5H*. Consequently, this strengthens the cotton plants’ resistance to *Verticillium* wilt, a disease induced by *Verticillium dahlia* (Guo et al., 2016). Furthermore, according to Li et al. (2020) reported that the enhancement of rice disease resistance is attributed to the thickening of sclerenchyma cells near the epidermis due to *OsMYB30*-induced lignin accumulation, which inhibits pathogen penetration and augments rice immunity. In this study, the expression of transcription factors such as *MYBP, AP2, MYBP, WRKY33, WRKY22*, and *ERF1* was activated after treatment with MT-DZY6715, which induced the expression of genes associated with the secondary metabolic pathways in *C. oleifera*, including *PAL*, *CAL*, *Laccase*, *HCT*, and *CHI*. Furthermore, the expression of these genes activated the secondary metabolic pathways, leading to the accumulation of secondary metabolites and ultimately enhancing the disease resistance of *C. oleifera*.

Lignin biosynthesis is a core branch of plant secondary metabolism, with its foundation rooted in three primary hydroxycinnamyl alcohols: p-coumaryl alcohol, coniferyl alcohol, and sinapyl alcohol (Yao et al., 2021). These alcohols undergo radical coupling reactions to synthesize lignin. During the process of lignin biosynthesis, phenylalanine ammonia-lyase (PAL), 4-coumarate:CoA ligase (4CL), and cinnamate 4-hydroxylase (C4H) play crucial roles (Ma et al., 2023). The process initiates with phenylalanine, which is converted into cinnamic acid under the action of PAL. Subsequently, cinnamic acid undergoes hydroxylation catalyzed by C4H, producing *p*-coumaric acid (Yao et al., 2021). Then, *p*-coumaric acid is hydroxylated at C3 position catalyzed by coumarate 3-hydroxylase (C3H), generating caffeic acid. The hydroxyl group at the C3 position of caffeic acid is further methylated by cinnamyl alcohol dehydrogenase (COMT), resulting in the production of ferulic acid (Choi et al., 2023; Marchiosi et al., 2020). This series of transformations provides the necessary intermediates for subsequent lignin monomer synthesis. Next, *p*-coumaric acid and ferulic acid undergo consecutive catalysis by enzymes, including 4CL, cinnamoyl-CoA reductase (CCR), cinnamyl alcohol dehydrogenase (CAD), quinate/shikimate p-hydroxycinnamoyltransferase (HCT), p-coumaroylshikimate 3’-hydroxylase (C3’H), and caffeoyl-CoA O-methyltransferase (CCoAOMT), forming monolignols—the precursors of lignin (Liu et al., 2021; Zhao et al., 2022a). Finally, these monolignol precursors, catalyzed by lignin-forming enzymes, particularly peroxidases (PRX) and laccases (LACs), undergo polymerization through radical reactions on the cell wall, resulting in the formation of lignin (Blaschek and Pesquet, 2021; Jalal et al., 2025; Peracchi et al., 2024). This process enhances the stability and mechanical strength of plant cell walls, thereby improving the plant’s resistance to external environmental and biological stresses (Han et al., 2022; Yadav and Chattopadhyay, 2023). In our research, compared to the control group, the application of MT-DZY 6715 significantly increased the activity of PAL, C4H, 4CL, CAD, and laccase. This synergistic enhancement of these enzyme activities that associated with disease resistance defense, promoted the synthesis and accumulation of lignin, thereby effectively enhanced the resistance of *C. oleifera* to anthracnose.

The plant cell wall, comprising intricate components such as cellulose, hemicellulose, and pectin, stands as a formidable physical barrier, crucial for safeguarding plants against pathogen invasion (Lorrai and Ferrari, 2021). Within this structure, cellulose molecules coalesce into microfibrils, forming the foundational scaffold of the cell wall. Pectin, on the other hand, plays a pivotal role in maintaining the structural integrity and mechanical properties of the cell wall, while also participating in plant defense mechanisms by modulating cell wall permeability and signaling pathways (Bidhendi and Geitmann, 2016). Lakshmesha et al. (2005) reported that the decreased activities of cellulase and pectinase hindered the degradation of cell walls, and effectively delaying the spread of anthracnose on pepper fruits. Similarly, Li et al. (2018) found that intercropping potato onion with tomato stimulates the roots to reduce the activities of cellulase and pectinase, thereby inhibiting the growth of the soil-borne pathogen *Verticillium dahliae* and enhancing tomato’s resistance to *Verticillium* wilt. According to Xiang et al. (2025), MT effectively enhanced mango resistance to anthracnose by inducing the expression of the IF *MiWRKY45*, which in turn activated the phenylpropanoid metabolic pathway, and promoted the synthesis of lignin and other defense compounds. Similarly, research by Sun et al. (2021) suggested that to a single treatment, the combined application of MT and *Meyerozyma guilliermondii* Y-1 markedly hinders the invasion and spread of *Botrytis cinerea* by enhancing defense-related enzyme activities, such as POD, PAL and PPO, as well as accumulating total phenolics and lignin content. Consequently, this combination fortifies the mechanical strength of fruit cell walls, thereby enhancing apple fruit resistance to gray mold disease. According to our experimental results, treatment with MT-DZY 6715 effectively inhibited both cellulase and pectinase activities, maintaining elevated levels of cellulose and protopectin in the leaves of *C. oleifera*, which better preserved the mechanical properties of *C. oleifera* cell walls and ultimately enhanced its disease resistance.

## 5 Conclusion

In summary, the application of MT-DZY 6715 effectively inhibits the growth of *C. siamense*, consequently decreasing the incidence of *C. oleifera* anthracnose. The structural characteristics of *C. oleifera* leaves were fortified, resulting in an elevated disease resistance, after being treated with MT-DZY 6715. Additionally, MT-DZY 6715 treatment stimulates the expression of genes associated with secondary metabolic pathways, increasing the activities of enzymes like PAL, 4AL, C4H, and CAD, while simultaneously suppressing the activities of cellulase and pectinase. These combined effects synergistically promote the accumulation of secondary metabolites, maintain the mechanical integrity of cell walls, and enhance the resistance of *C. oleifera* against anthracnose. Therefore, MT-DZY 6715 emerges as a promising biological control agent for managing *C. oleifera* anthracnose. However, future research is still needed to comprehensively evaluate the long-term prevention and control effects of MT-DZY 6715 under different environmental conditions through field trials. Additionally, the molecular mechanisms underlying the interaction between MT-DZY 6715 and *C. oleifera* should be explored in depth using transcriptomics and proteomics. Furthermore, exploring potential synergistic effects between MT-DZY 6715 and other biocontrol agents or chemical pesticides to enhance the integrated disease control efficacy. Collectively, these future research paths hold significant potential for promoting the sustainable management of *C. oleifera* anthracnose, offering novel technological support and solutions for the healthy development of the *C. oleifera* industry.

## Data Availability

The transcriptome data presented in this study have been deposited in the NCBI Sequence Read Archive (SRA) with the accession number PRJNA1268391.
