# Supplementary material for: Melatonin-induced Bacillus tequilensis enhanced the disease resistance of Camellia oleifera against anthracnose by modulating cell wall and phenylpropanoid metabolism
Source: Front Plant Sci. 2025 Jun 9;16:1593369. doi: 10.3389/fpls.2025.1593369 (PMC12183246; doi:10.3389/fpls.2025.1593369)
Supplement: Supplementary file 1 [file Table1.docx]

Table S1 The information of DEGs and associated primers for conducting qRT-PCRanalysis

| Gene | ID | Primer sequence |
| --- | --- | --- |
| *GST*-F | maker-HiC_scaffold_15-snap-gene-706.11 | GCAGATGGTGTTGTGAAGGATATTATG |
| *GST*-R |  | CTGGTGTATAGAGGCACTTCTAATTGG |
| *HCT*-F | snap_masked-HiC_scaffold_12-processed-gene-1939.19 | CGATTCATGACGCGGATTTT |
| *HCT*-R |  | ATTGGTTGGACTCGGCAATG |
| *MYBP*-F | maker-HiC_scaffold_1-snap-gene-2153.40 | TTCTCAATGTCAAGGAACGGTAGC |
| *MYBP*-R |  | TCTGTACCAACAAGTAATGATCCTCTG |
| *UGT72E*-F | augustus_masked-HiC_scaffold_10-processed-gene-1874.40 | CGATCAAAATGAACCCCTCA |
| *UGT72E*-R |  | TATTCGATCCCTATGCGCAC |
| *PE*-F | genemark-HiC_scaffold_12-processed-gene-430.32 | TTGCTAAGACGACACGAAACAGAC |
| *PE*-R |  | CGGCACTTGGCGATGATGAC |
| *WRKY22*-F | maker-HiC_scaffold_12-snap-gene-890.38 | CCGGTGACTGAAAAGATGGA |
| *WRKY22*-R |  | CACGGAAAAGTCAAACTCGC |
| *Actin*-F | / | CATCCCTCAGCACCTTCC |
| *Actin*-R |  | CCAACCTTAGCACTTCTCC |
